# Remote sensing continuity: a comparison of HTP platforms and potential challenges with field applications

**DOI:** 10.3389/fpls.2023.1233892

**Published:** 2023-09-18

**Authors:** Andrew W. Herr, Arron H. Carter

**Affiliations:** Department of Crop and Soil Sciences, Washington State University, Pullman, WA, United States

**Keywords:** high throughput phenotyping, plant breeding, spectral reflectance indices, unoccupied aircraft system - UAS, field based phenotyping, remote sensing

## Abstract

In an era of climate change and increased environmental variability, breeders are looking for tools to maintain and increase genetic gain and overall efficiency. In recent years the field of high throughput phenotyping (HTP) has received increased attention as an option to meet this need. There are many platform options in HTP, but ground-based handheld and remote aerial systems are two popular options. While many HTP setups have similar specifications, it is not always clear if data from different systems can be treated interchangeably. In this research, we evaluated two handheld radiometer platforms, Cropscan MSR16R and Spectra Vista Corp (SVC) HR-1024i, as well as a UAS-based system with a Sentera Quad Multispectral Sensor. Each handheld radiometer was used for two years simultaneously with the unoccupied aircraft systems (UAS) in collecting winter wheat breeding trials between 2018-2021. Spectral reflectance indices (SRI) were calculated for each system. SRI heritability and correlation were analyzed in evaluating the platform and SRI usability for breeding applications. Correlations of SRIs were low against UAS SRI and grain yield while using the Cropscan system in 2018 and 2019. Dissimilarly, the SVC system in 2020 and 2021 produced moderate correlations across UAS SRI and grain yield. UAS SRI were consistently more heritable, with broad-sense heritability ranging from 0.58 to 0.80. Data standardization and collection windows are important to consider in ensuring reliable data. Furthermore, practical aspects and best practices for these HTP platforms, relative to applied breeding applications, are highlighted and discussed. The findings of this study can be a framework to build upon when considering the implementation of HTP technology in an applied breeding program.

## 1 Introduction

Severe weather and climate change are creating new challenges in maintaining and improving global food production. Plant breeding is an important tool in adapting to these difficulties (Brown et al., 2015). However, the plant breeding process is not immune to extreme or unpredictable environmental conditions, impacting selection efficiency and genetic gain (Xiong et al., 2022). Despite steady increases in genetic gain, global cereal crop demand is projected to surpass production by 2050 (Ray et al., 2013). A practical method for increasing crop production is through the release of new cultivars by increasing genetic gain. On average, it takes 7-12 years to release a new winter wheat cultivar (Carver, 2009). Thus, it is imperative to look at new methods that increase genetic gain, decrease cycle time, and improve grain yield in an era of climate change and extreme environmental variables (Xiong et al., 2022). Recent advancements in genomic technologies have provided breeders with large amounts of data to utilize genomic and marker-assisted selections. However, genomic data has limited use without the backing of phenotypic data, creating a new bottleneck in the industry, and limiting cultivar development efficiency (Mir et al., 2019). One proposed solution to this limitation is implementing high throughput phenotyping (HTP) methods associated with established breeding strategies (Reynolds et al., 2020).

The development of HTP results from advancements in imaging sensors, image processing technology, and an understanding of secondary phenotypic traits (Pauli et al., 2016; Mir et al., 2019). Despite these advancements, unoccupied aircraft systems (UAS) technology can be fastidious and resource intensive for simple plant breeding applications. Most HTP strategies require the purchase of expensive specialized equipment and tedious data standardization and processing pipelines. There is a continued need to identify and adapt HTP technology to better aid the breeder while maintaining cost-effectiveness (Reynolds et al., 2020).

There are three primary options in field-based HTP. Satellites allow for the high throughput collection of field-scale images yet are limited by image frequencies and resolution, critical factors in plot-level research applications. Alternatively, ground-based and handheld systems provide high resolution imaging across a wide range of frequencies but can be challenging to handle and capture larger-scale, multi-plot images quickly. UAS provide a “goldilocks” ratio of utility, temporal frequency, and spatial resolution (Araus and Cairns, 2014; Song et al., 2021). Rotocopters are a versatile platform that allows for high throughput, high resolution image capture. Due to power usage and battery capacity, the limitations of the platform arise in payload capacity and flight time (Sankaran et al., 2015b; Xie and Yang, 2020).

The spectral reflectance data collected from handheld radiometers and UAS cameras has minimal uses in its raw form. Spectral reflectance indices (SRI) are used to evaluate target features and remove image noise creating a practical, standardized trait value (Myneni et al., 1995; Xue and Su, 2017). Vegetation indices are developed by evaluating the reflectance value of the plant canopy at specific light bands associated with photosynthetic mechanisms. Normalized Difference Vegetation Index (NDVI) is a prevalent index used to evaluate plant health by evaluating contrast in the maximum absorption of red in the leaf through chlorophyll pigmentation and the maximum reflectance of near-infrared due to leaf cellular structure (Rouse, 1974). Normalized Difference Red-edge Index (NDRE), another popular standard vegetation index, works similarly to NDVI but replaces red with red-edge absorption relative to NIR (Gitelson and Merzlyak, 1996). The vegetation index used depends on the crop, growth stage, and target trait. These factors influence reflectance values and relative index effectiveness (Wientjes et al., 2017; Lozada et al., 2020; Herr et al., 2023). Vegetation indices have many applications in capturing routine trait estimates like plot quality, biotic, and abiotic stress (Sankaran et al., 2015a; Guo et al., 2021; Sarkar et al., 2022; Sapkota et al., 2023), as well as previously infeasible traits like chlorophyll content and nitrogen content (Xie and Yang, 2020; Yin et al., 2022). Unlike vegetation indices, water indices such as Normalized Water Index (NWI) use infrared range reflectance to evaluate stomatal conductance and overall photosynthetic efficiency (Babar et al., 2006). Water indices can evaluate and predict relative water content, leaf osmotic potential, stomatal conductance, and canopy temperature(Gutierrez et al., 2010; Bal et al., 2021; Visitacion et al., 2022).

For most breeders working with cereal crops, grain yield is the critical trait of interest and an ideal gauge of overall biological and economic performance. Grain yield in wheat is highly quantitative and can make selection efficiency difficult (Reynolds et al., 2012). It is well established that NDVI and other vegetation indices like NDRE and NWI, through high throughput multispectral imaging, correlate with cereal crop grain yields (Geipel et al., 2014; Gracia-Romero et al., 2017; Lozada et al., 2020). It has also been shown that SRI data can be utilized to improve tools like genomic selection for grain yield. Thus, grain yield is an appealing trait for a breeder to focus on when implementing HTP approaches (Reynolds et al., 2020; Montesinos López et al., 2022; Herr et al., 2023).

SRI heritability and correlation to grain yield are leading indicators of SRI and platform utility. A strong repeatable relationship to grain yield can determine data quality and efficiency of selection in large-scale field-based breeding applications. While the sensors evaluated in this study collected the same SRIs, each has a different manufacturer, sensor type, and processing pipeline. Sensor differences create the potential for variances in data value and quality. These variances are compared and discussed. Finally, the practical aspects of the platforms are compared for their potential cost relative to the benefit they could provide (i.e., the improved resolution of ground collected data is not worth the extra logistics required in data collection).

With the growing interest in utilizing high throughput phenotyping technology in plant breeding, this study aimed to compare the SRI data collected from breeding trials between ground systems and UAS and determine if the use of ground-based handheld systems provides an increased resolution and data quality that justify the negative aspects of the platform, like collection time, data noise, and secondary applications.

## 2 Materials and methods

### 2.1 Study population

**Figure 1:**
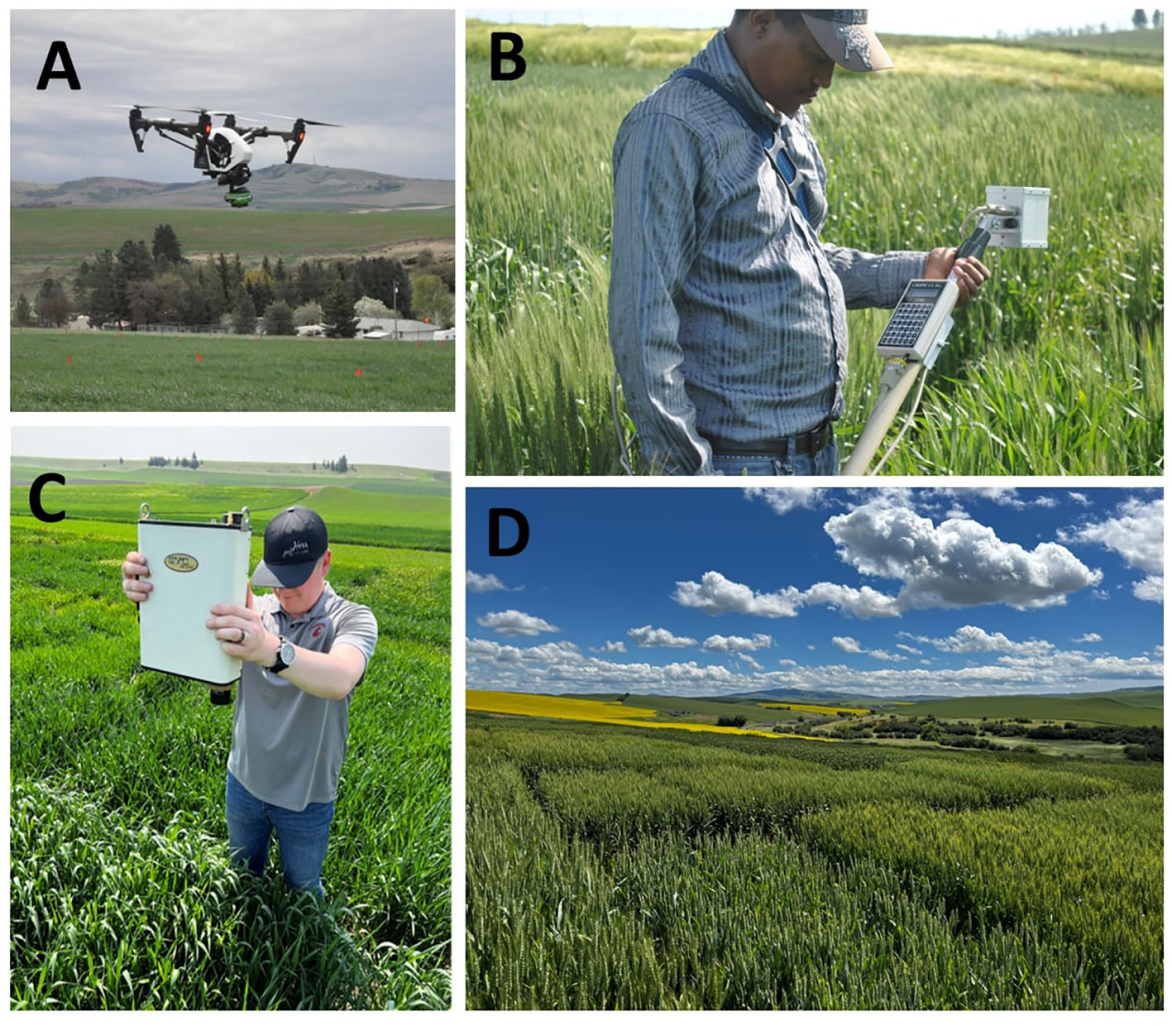
The imaging systems used in this study were **(A)** a DJI Inspire 1 UAS with a Sentera Quad Multispectral Sensor, **(B)** Cropscan MSR16R, and **(C)** SVC HR-1024i. **(D)** highlights the study location.

The Washington State University (WSU) winter wheat breeding program has collected multispectral data with three different phenotyping systems as indicated in **Figure 1**: A handheld multispectral radiometer, the Cropscan MSR16R (CROPSCAN Inc., Rochester, MN, USA), a handheld full-range hyperspectral spectro-radiometer, the Spectra Vista Corporation (SVC) HR-1024i (Spectra Vista Corporation, Poughkeepsie, NY, USA), and a UAS-based system, a Sentera Quad Multispectral Sensor (Sentera Inc., Minneapolis, MN, USA) mounted on a DJI Inspire 1 rotor copter platform.

Data for all platforms was collected at anthesis due to its established relationship with grain yield (Duan et al., 2017; Lozada et al., 2020). Each population evaluated was sampled on the same day by both the UAS and compared handheld system. Handheld data was collected within a six-hour window of solar noon, which was the typical time required to collect data given the number of plots in the trials. UAS data was collected within a four-hour window of solar noon. In these trials, UAS data was often collected at or near solar noon to try and be in the middle of the handheld data collection timeframe, and flights often took 20 minutes. The UAS mounted with the Sentera camera flew a programmed route at an altitude of 45 m, with an 85% longitudinal and lateral overlap of georeferenced images. All data was collected on days with clear skies to limit variability in solar radiation. All trials were grown in Pullman, WA, as shown in **Figure 1**, and include:

- A genetically diverse Quality Association Mapping (QAM) panel;
- Unreplicated single plot yield trials of soft white and hard red winter wheat;
- Replicated alpha-lattice preliminary yield trials of soft white and hard red winter wheat;
- Replicated alpha-lattice advanced yield trials of soft white and hard red winter wheat.

**Table 1** outlines the study populations’ characteristics, including year, trial design, total number of unique entries, number of total plot observations, and HTP data type collected. Plots were planted using a double-disc 8-row small plot planter at a seed density of 250 seed per square meter. Total plot size was 1.5 meter wide by 3.5 meter long. Ground was prepared by grower cooperators using minimum-tillage techniques and practices customary of the region. Grain yield data were collected at all locations with a Zurn 150 harvester (Zurn Harvesting GmbH & Co. KG, Waldenburg, Germany). Weather data for each year can be found at https://weather.wsu.edu/ for the Pullman, WA location. Single environment adjusted means were calculated for all observations of grain yield. Grain yield was the focus of observation given its importance as the final end-value selection parameter in many plant breeding programs.

**Table 1:** Study populations for HTP platform comparison.

Trial	Year	Design	Replication	Number of Entries	Total Observations	UAS	Cropscan	SVC
Single Plot	2018	Augmented Design with repeating checks	1	1438	1503	X	X	
Preliminary	2018	Alpha-Lattice	3	168	504	X	X	
QAM	2018	Augmented Design with repeating checks	1	480	528	X	X	
QAM	2019	Augmented Design with repeating checks	1	480	528	X	X	
Single Plot	2020	Augmented Design with repeating checks	1	178	195	X		X
Advanced	2020	Alpha-Lattice	3	48	144	X		X
Single Plot	2021	Augmented Design with repeating checks	1	213	227	X		X
Preliminary	2021	Alpha-Lattice	3	54	162	X		X
Advanced	2021	Alpha-Lattice	3	46	138	X		X

QAM, Quality Association Mapping Panel; X, Indicates HTP Data Type Collected.

### 2.2 UAS phenotypic data

The Sentera Quad Multispectral Sensor covered target bands of interest for winter wheat evaluation. The camera has four sensors that cover eight broad spectral bands between 450 nm and 970 nm. Collected UAS images are stitched and prepared for data extraction in Pix4Dmapper (Pix4D Inc., Denver, CO, USA), creating a single orthomosaic image for each sensor per location. Orthomosaic images were transferred to Quantum Geographic Information System (QGIS) for plot identification and then further processed with a custom R code for calibration, index calculation, and single plot mean data extraction. In 2018 and 2019, a single reflectance panel (85% reflectance) was used for radiometric calibration on RBG and red edge bands. Quantum efficiency coefficients were used to calculate NIR using:


NIR=(2.921×Blue)−(0.754×Red).

The NIR band was then normalized with a coefficient of 3.07 during the calculation of SRIs (Ortiz et al., 2021). In 2020 and 2021, a set of calibration panels (five panels ranging from 2% – 85% reflectance, MosaicMill Oy, Vantaa, Finland) was implemented. Iqbal et al. (2018) developed a simple radiometric calibration methodology using a set of calibration panels with a known variation of reflectance at each broadband wavelength of interest. The band layers are adjusted based on the relationship:


SR=DN×m±b,

where digital numbers (DN) are the raw observed pixel values for collected orthomosaic images and Surface Reflectance (SR) is the true reflectance value. Slope (m) and intercept (b) are variables explaining the relationship between observed and true values of the reflectance panels. Once slope and intercept are calculated based on the reflectance panels’ regression, the corresponding bands can be adjusted.

### 2.3 Ground phenotypic data

Like the Sentera sensor, the CROPSCAN MSR16R covers target bands of interest. The CROPSCAN radiometer has 16 broad spectral bands that range from 430 nm to 970 nm. Before data collection the sensor is calibrated using manufacture provided calibration panel. The sensor was attached to a pole and placed 1m directly above the wheat canopy in the middle of the plot. One plot is collected at a time, and a mean value for each spectral band is logged for that plot. An irradiance light sensor accounts for light variation and reduces noise in reflectance values. The CROPSCAN MSR system software is used to retrieve collected band values. Plot reflectance values were normalized across all observations by dividing each plot reflectance value by the standard deviation of reflectance values within a trial.

The SVC HR-1024i is a hyperspectral sensor collecting thousands of narrow band values between 338 nm and 2515 nm for each plot. Before sampling, the sensor was calibrated using the manufacture provided calibration panel. The SVC was held by hand at a height of 0.75 m above the plot at an approximate 20-degree angle. Collected SVC data reflectance curves were observed for each plot. Observations with abnormal reflectance curves below 1000 nm were removed from the evaluation. Band reflectance values were normalized as done with CROPSCAN data. Broadband values that reciprocate the collected UAS bands were then calculated by averaging all SVC narrowband values within the 50 nm desired broadband window.

### 2.4 Spectral reflectance indices calculation and data analysis

Comparing these systems is based on three overlying factors: grain yield correlation with water indices, grain yield correlation with vegetation indices, and overall utility for a large-scale breeding program. The processed spectral reflectance data collected from each plot for both the handheld and UAS platforms were used to calculate the vegetation indices NDVI, NDRE, Transformed Chlorophyll Absorption Reflectance Index (TCARI), Modified Triangular Vegetation Index (MTVI), and the water index, NWI. NDVI and NWI are the most commonly used in each of their corresponding categories and are ideal measures of plant stress and canopy water stress, respectively, in winter wheat (Prasad et al., 2007; Lozada et al., 2020). NDRE, TCARI and MTVI were chosen because of their past success in our breeding program in correlating to yield and accounting for environmental variability. The spectral reflectance bands used and formulas for these indices are shown in **Table 2**.

**Table 2:** Spectral reflectance index equations.

Spectral Reflectance Index	Abbreviation	Equation	Reference
Normalized Difference Vegetation Index	NDVI	(R800−R680)/(R800+R680)	(Rouse et al., 1974)
Normalized Difference Red Edge	NDRE	(R800−R700)/(R800+R700)	(Gitelson and Merzlyak, 1996)
Transformed Chlorophyll Absorption Reflectance Index	TCARI	3×[(R700−680)−0.2×(R700−R550)(R700/R680)]	(Haboudane et al., 2002)
Normalized Water Index	NWI	(R970−R800)/(R970+R800)	(Gao, 1996)
Modified Triangular Vegetation Index	MTVI	((R700−R550))/√((2*R800+1)^2−(6*R800−5*√R680)−0.5))	(Haboudane et al., 2004)

Broad-sense heritability (H2) was calculated for SRIs across all sampled locations for grain yield. Genotype, replication, block, environment, and genotype by environment variation were used as random effects in the calculation of H2 with the formula:


$$H^{2}=\frac{\sigma_{G}^{2}}{\sigma_{G}^{2}+\frac{\sigma_{GE}^{2}}{x}+\frac{\sigma_{\epsilon }^{2}}{xr}}$$

where $\sigma_{G}^{2}$ is genetic variance, $\sigma_{GE}^{2}$ is variation due to genotype by environmental effect, $\sigma_{\epsilon }^{2}$ represents variation due to error, *x* signifies the number of environments, and *r* represents the number of replications (Bernardo, 2002). Variance components used in heritability calculations were estimated using the “lme4” package in R. Heritability, in conjunction with correlation to grain yield, will indicate an index’s success in indirect selection.

Phenotypic correlations among traits were calculated within the two datasets as Pearson correlations using “cor’ function in R. Scatterplots and regressions for each unique year and platform combination was generated using “ggplot2” in R.

Principal component analysis (PCA) was conducted for the 2018-2019 and 2020-2021 populations using NDVI, NDRE, TCARI, MTVI and NWI for each platform as well as grain yield. PCA was conducted using the “FactoMineR” package in R.

## 3 Results

### 3.1 SRI correlation across platforms and with grain yield

**Figure 2:**
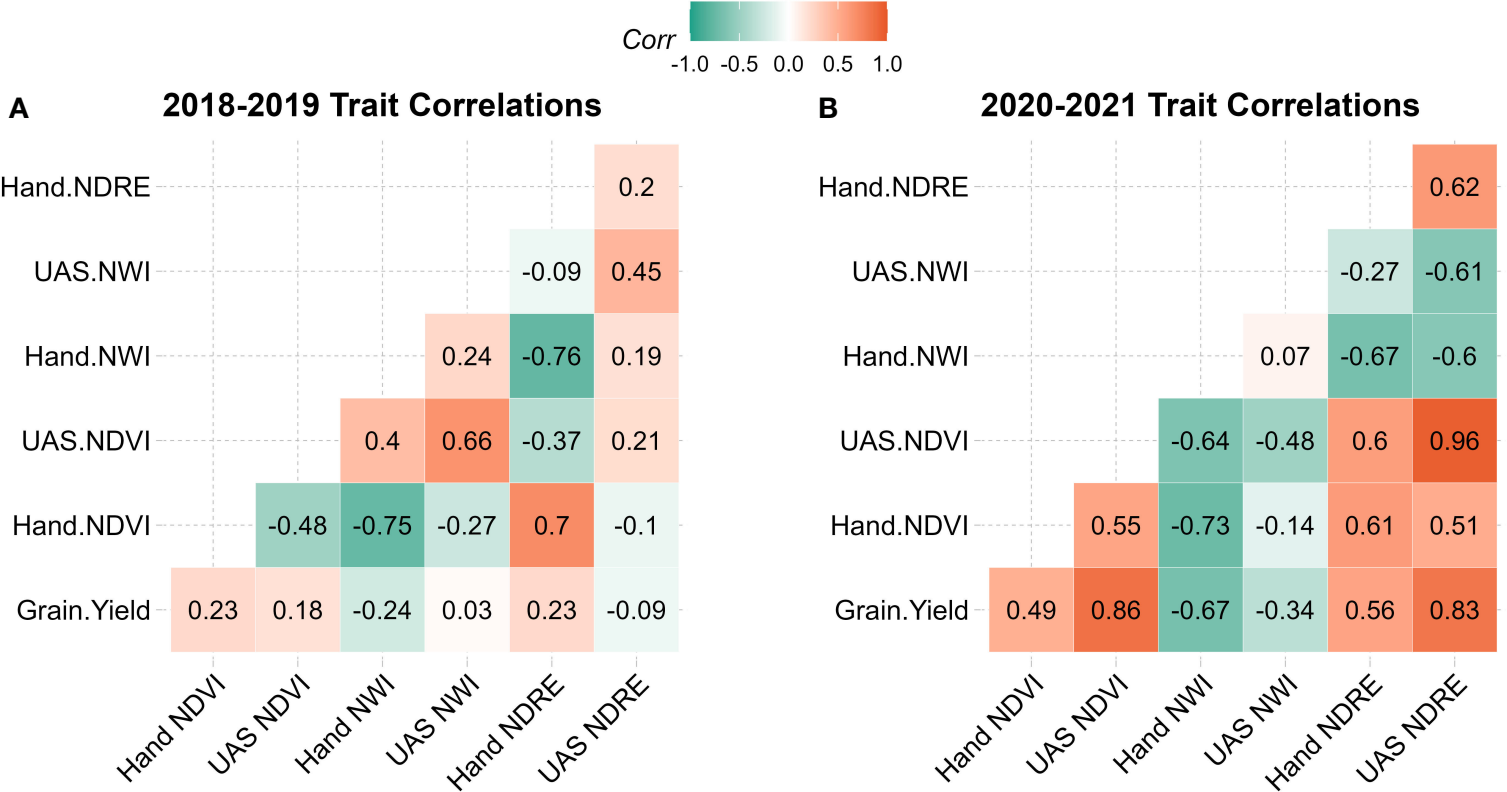
Correlation table of grain yield, handheld collected SRIs, and UAS collected SRIs in **(A)** 2018-2019 population and **(B)** 2020-2021 population. NDVI, Normalized Difference Vegetation Index; NDRE, Normalized Difference Red Edge; NWI, Normalized Water Index.

**Figure 3:**
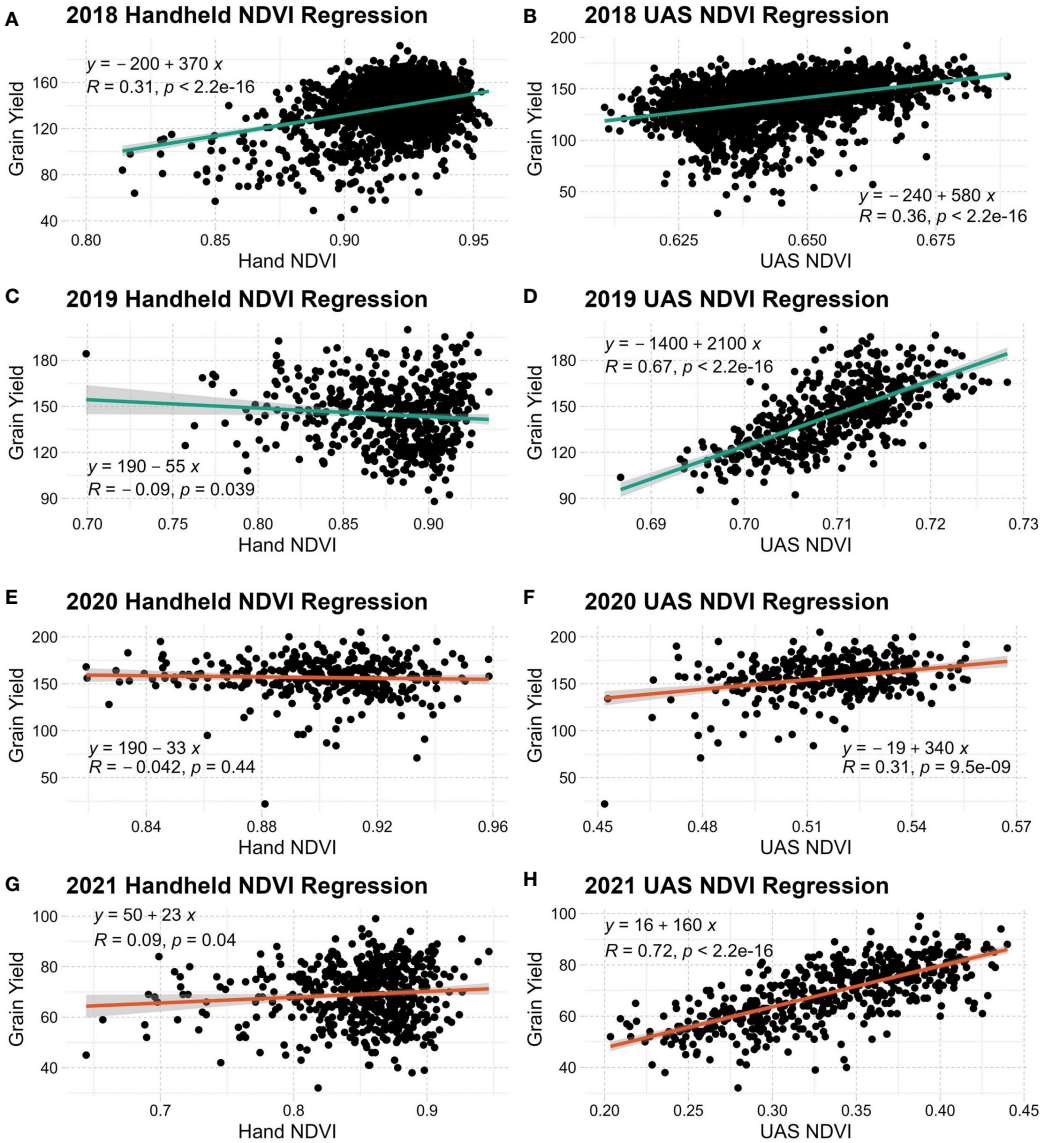
Linear regression of grain yield and handheld NDVI **(A, C, E, G)** or UAS NDVI **(B, D, F, H)** in each year evaluated. NDVI, Normalized Difference Vegetation Index.

Within each population, the correlation of indices was evaluated between collection methods as well as between indices and grain yield. In 2018-2019 correlations with grain yield were close to zero or slightly negative with handheld NWI, ranging from -0.23 to 0.22. Handheld NDVI was negatively associated with UAS NDVI with a correlation of -0.48, whereas handheld NWI and NDRE had a small positive association with UAS with a correlation of 0.24 and 0.2, respectively. In the 2020-2021 population, NDVI and NDRE had a moderate to high positive correlation between collection strategies and grain yield, as seen in **Figure 2** and reinforced by PCA in **Figure 3**. NWI had a low correlation between UAS and handheld, with a coefficient of 0.07. When correlated to grain yield, UAS and handheld NWI had a negative association of -0.34 and -0.67, respectively. The negative correlation between water-based indices and grain yield was supported in PCA. This negative correlation is expected in NWI with both vegetation indices and grain yield. A lower NWI value indicates higher water content in crop canopy.

### 3.2 Linear relationship of SRIs to grain yield

A clearer relationship can be seen when linear regressions are conducted with SRIs and grain yield, as seen in **Figure 3**. Across all years and environmental conditions, UAS NDVI has an expected linear distribution relative to grain yield. Both handheld systems used in this study produced high NDVI values across observations while producing more non-normal distributions relative to grain yield. UAS NDVI generally has a stronger linear relationship to grain yield over the handheld counterpart. Only NDVI is shown because of its relevance to wheat. All other SRIs evaluated have similar trends.

### 3.3 PCA of SRIs across platforms and grain yield

**Figure 4:**
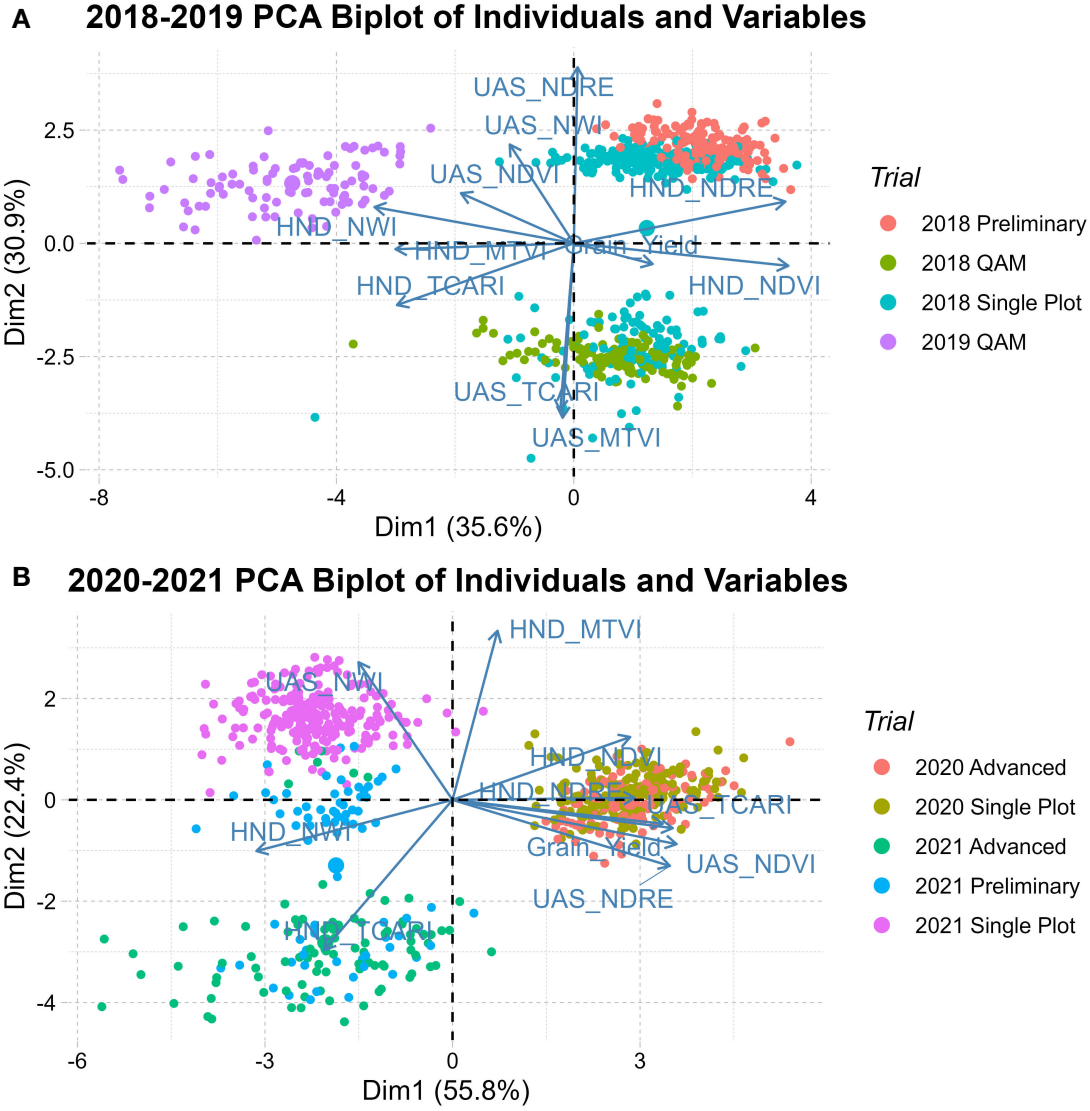
Principal component biplot of individuals and vector of variables in **(A)** 2018-2019 population and **(B)** 2020-2021 population showing the genetic relationships of QAM diversity panel, early generation single plot, preliminary, and advanced trials.

The first principal component (Dim1) captured between 35.6% and 55.8% of the phenotypic variation. The second principal component (Dim2) was only able to explain between 30.9% and 22.4% of trait variation. PCA biplots of individuals in both populations group years along Dim1, indicating strong between-year environmental variability. QAM diversity panels group closely within their given year, while breeding trials (single plot, preliminary, and advanced) tend to spread across Dim2. As seen in **Figure 4**, the 2020 observations are tightly grouped due to ideal growing conditions reducing genetic expression in trait variability. In 2018-2019 handheld generated indices contributed most in Dim1 while UAS generated indices contributed more in Dim2. This differs from 2020-2021 where most indices were contributing to Dim1 whereas only handheld MTVI and UAS NWI were major contributors to Dim2.

### 3.4 Heritability of grain yield and SRIs in evaluated trials

Broad-sense heritability for spectral indices of all years evaluated was moderate to high, with a range of 0.50 to 0.80. Grain yield heritability was also calculated at 0.65 in 2018-2019 and 0.76 in 2020-2021, as seen in **Table 3**. Across both populations, UAS collected indices had a higher heritability than handheld collected indices. This difference was greater with NDVI and NDRE than with NWI.

**Table 3:** Broad-sense heritability (H^2^) of grain yield, UAS indices, and handheld indices.

Population	Grain Yield	Handheld NDVI	UAS NDVI	Handheld NWI	UAS NWI	Handheld NDRE	UAS NDRE
2018-2019	0.65	0.52	0.80	0.50	0.58	0.50	0.60
2020-2021	0.76	0.55	0.68	0.62	0.70	0.58	0.67

## 4 Discussion

In this study, we have outlined the differences in the correlation and heritability performance of SRIs collected from handheld and UAS systems relative to grain yield. This study evaluated HTP data of a breeding population from a single location, over four highly differing years. This is typical of most breeding programs where lines are evaluated initially and selected based on performance at one location. Despite these factors, there are clear differences in the capability of the tested HTP systems for application in a breeding pipeline to improve grain yield selection potential as secondary traits. Both phenotypic correlation and heritability of SRIs were assessed to evaluate the utility in improving selection for grain yield. This section, along with the discussion of analytical results, will break down the less tangible aspects of the HTP systems used in this study and their relative potential utility in breeding applications.

### 4.1 SRI correlation and precision across platforms

The correlations of SRIs in the 2018-2019 dataset were generally lower than that of the 2020-2021 dataset. It is important to note that NWI is a water index that negatively associates with canopy water content. A higher NWI value indicates lower canopy water content, meaning that a strong negative correlation to grain yield is ideal (Bandyopadhyay et al., 2014). This relatively low correlation in 2018-2019 is potentially due to inadequate data quality caused by more primitive data standardization and poor sensor quality, where only one calibration panel was used. The poor data quality in 2018-2019 is also exemplified in the low correlations of corresponding SRIs between handheld and UAS. Similar findings were shown by Deng et al. (2018) and Díaz-Delgado et al. (2019), both highlighting inconsistencies in sensor performance and correlation, especially when sensor quality or calibration methods are inadequate. Finally, despite moderate heritability, the 2018-2019 SRI data correlates poorly with grain yield. This suggests that the collected data was not capturing the chlorophyll or water content targeted by SRIs, possibly because of the reduced calibration panel set. Ensuring that data collected is of the highest quality is always essential. As additional research was published suggesting the move from a single calibration panel to multiple panels, subsequent data was improved and yielded higher correlations with grain yield (Duan et al., 2017; Guo et al., 2019).

Unlike the 2018-2019 dataset, in 2020 and 2021, correlations are improved to moderate or high across SRI and platform. UAS data correlations are most likely improved over the 2018-2019 population due to an improved image calibration strategy using a set of five calibration panels. The 2020-2021 data also displays expected patterns across the correlation table between grain yield, handheld and UAS data indicating a more successful capture of target physiological characteristics relative to the 2018-2019 dataset. There are generally stronger correlations among grain yield UAS data in 2020-2021 relative to 2018-2019. These differences between datasets collected by the two ground based systems could possibly be because of variation in climatic conditions of the years. More likely, the improved correlations in 2020 and 2021 were because of improved data quality with the enhanced calibration strategies that were implemented.

The handheld radiometer systems do not have as quick of a collection speed as UAS, allowing for the introduction of error, similar results were found by Tattaris et al. (2016). This issue will be discussed in later sections. This study also validated Tattaris et al. (2016) in higher correlations of UAS derived vegetation indices to yield relative to ground based proximal sensors. In 2020 and 2021, handheld platforms did outperform UAS with NWI correlations. The outlier in correlation is most likely due to NWI’s susceptibility to environmental variability and general sensor quality. These results are corroborated in Gutierrez et al. (2010) and Bandyopadhyay et al. (2014), highlighting difficulties in working with NWI. The SVC sensor used in 2020 and 2021 is a hyperspectral sensor capable of greater precision in reflectance evaluations. Reflectance bands used in calculating NWI are within the median reflectance range of the SVC sensor, whereas the UAS camera works with secondary modified sensors.

### 4.2 SRI heritability and reliability in selection

Across all evaluated SRIs in both populations, UAS data produced a higher broad-sense heritability than handheld systems. This difference in heritability between systems is most likely due to the increased variability of SRI data introduced during a lengthened data collection window. Handheld systems have the disadvantage of collection efficiency, taking approximately 10 seconds per plot, whereas a UAS system can average under 2 seconds per plot. While the UAS, Cropscan and SVC all have methods for sensor calibration, slight changes in solar position and intensity likely impacted reflectance readings. It has been well established that minimization of error during the spectral reflectance data collection process is critical to the final data quality (Guo et al., 2016; Ortiz et al., 2021). Because the UAS system captures several plots at a time and the same plot multiple times, all within a 20-30 min window, it is likely that UAS reflectance data has a reduced potential for error relative to the handheld radiometers used. This difference in data quality is also observed in NDVI’s relationships to grain yield across years, shown in **Figure 3**.

The moderate SRI heritability observed in this study is expected due to a portion of the study population being unreplicated trials. We also expect heritability in the 2018-2019 population to be lower than the 2020-2021 population due to the increased genetic diversity of the population from the inclusion of the QAM diversity panel (Bowman et al., 2015). SRI heritability was generally lower than grain yield, limiting the potential application for indirect selection. However, the moderate correlation and heritability of SRIs suggest the potential for improved genetic gain when utilized as secondary traits in selection. The utilization of SRI data for utilization in breeding for grain yield is most promising when incorporated in genomic selection strategies as a covariate or in multivariate models as shown my Lozada et al. (2020) and Montesinos López et al. (2022) respectively.

### 4.3 Platform utility in a breeding program

For most plant breeding programs grain yield is the primary trait of interest. The highly quantitative nature of the trait can make selection and prediction efficiency difficult (Reynolds et al., 2012). There is evidence that SRI data can complement and improve tools like genomic selection and machine learning prediction for use in the breeding strategy of grain yield (Montesinos López et al., 2022; Herr et al., 2023). It is important to validate that the methods used in secondary trait data collection are high quality, heritable, and correlate well to the primary trait of interest.

The overarching goal of this study was to determine if the use of ground-based handheld systems provides an increased resolution and data quality that justify the negative aspects of the platform, like collection time, data noise, and secondary applications. As mentioned above, handheld systems have the disadvantage of collection speed; this difference is amplified when capturing large breeding trails. A UAS can collect all data of a 1000 plot breeding trial in approximately 30 min, whereas the handheld system will take roughly 3 hours. In smaller research programs and applications, this difference would have minimal impact on the ability to collect desired datasets. However, in large breeding programs with multiple trial locations, collecting reflectance data across all locations at more than one or two critical time points can be difficult. Solar and weather limitations create narrow windows for image capture, and UAS imaging allows for flexibility in data collection timing. Under ideal environmental conditions, UAS allows for quick data capture across several locations in a single day. The variability seen in heritability and correlation between handheld and UAS is partly due to the differential in capture time. The increased time it takes a handheld radiometer system to collect data on an entire breeding trial, 2-3 hours, creates the potential for changes in solar radiation caused by solar angle or cloud cover. This will produce within field errors in collected reflectance data, creating challenges in distinguishing genetic, phenotypic, and environmental variability (Tattaris et al., 2016).

The HTP systems used in this study highlight the reality of working with technology in long term breeding research applications. When first evaluating the potential of HTP, UAS sensors were not common, thus the Cropscan system was utilized as a platform that was easy to implement in a field-based breeding program. In 2018, as more UAS and sensors became available, they were used in tandem with the hand-held Cropscan. When the Cropscan broke in 2020, alternative solutions were pursued for a ground based radiometer, leading to the use of the SVC system. Similarly, with the UAS calibration, when starting in 2018 the manufacturer recommendations of a single white panel were used. As new research came out it became evident for the need to implement higher quality, multi-panel radiometric calibration in 2020 and 2021. With technology constantly changing and improving, it is important to recognize the potential improvements these can make. It also important to note that new methodology or equipment can impact the quality and reliability of SRI data as shown in this study. As other breeding programs begin using HTP, it is valuable to evaluate different UAS calibration strategies and handheld platforms within the same population and year, and across a diverse set of environments, to clearly identify each technology’s reliability.

Each of the three phenotyping systems used in this study has a different method of calibration, collection, and processing that influence the quality of data collected. The SVC and Cropscan radiometer systems initially use a white reference panel to calibrate the sensor. These radiometers do not collect actual images but a range of mean reflectance bands within the sensor field of view. The Cropscan system requires custom software for post-processing to populate reflectance values, and the SVC requires normalization and conversion of narrow hyperspectral band values to multispectral broadbands. A major disadvantage of these radiometers is their inability to screen for reflectance noise within the sensor field of view. The UAS used in this study collects images which are later stitched into an orthomosaic containing the desired reflectance values. These orthomosaics can be used to create soil masks, removing soil and other non-plant reflectance in calculating mean plot reflectance for later use in SRI calculation.

Calibration is another strategy for minimizing reflectance noise and standardizing collected data. All platforms in 2018-2019 and the handheld system in 2020-2021 used a simple single-panel radiometric calibration technique that utilizes the know reflectance of a white panel to adjust sensor readings based on the observed panel reflectance. This method is effective but is more limited in accurately adjusting each reflectance band (Iqbal et al., 2018). Radiometric calibration with a range of calibration panels, a method used for 2020-2021 UAS data collection, improves the former strategy by utilizing three to five reflectance panels with a known range of solar absorption. The range of panels can be used to produce a regression of expected reflectance against observed for each reflectance band of interest. This technique allows for more precise adjustment in individual band readings, producing more reliable reflectance values (Wang and Myint, 2015; Iqbal et al., 2018). The removal of soil noise, robust radiometric calibration, and short flight times minimize the error in the data collected, ensuring reflectance data quality across time and locations.

It is important to maximize limited resources in large-scale applied plant breeding research. Any implementation of HTP can be costly and time consuming. Because of this, when looking at implementing HTP into a breeding program, it is important to consider the versatility and range of the platform selected. The handheld radiometers used in this study can collect high resolution reflectance data across a broad spectral range with the potential for producing SRIs with moderate heritability and correlation to yield, yet are limited in their ability to account for soil noise or inconsistent solar radiation. The data capture speed limits the quality of data collection across locations and time. The lack of orthomosaic image capture in these systems also limits access to secondary traits of interest like plant height and canopy coverage estimates.

With the continual improvement in technology and software, the barrier to entry for UAS phenotyping continues to drop. The speed and efficiency of UAS minimizes labor and cost while providing quality data for further use in breeding strategies. However, it is important to consider best practices that will minimize unwanted environmental variability in collected UAS data. One way that this can be done is by utilizing multiple radiometric calibration panels as outlined in 2020 and 2021 UAS data collection (Iqbal et al., 2018). Another practice that can minimize unwanted variability is in timing of UAS flights. Most sensors utilized on a UAS are passive sensors, therefore, it is important to adjust for shadowing and variability of solar radiation. For best results it is recommended to fly within a 4-5 hour window of solar noon on days without clouds (Ortiz et al., 2021). In plant breeding programs looking to incorporate high throughput phenotyping, the UAS is an efficient and versatile option that when used properly can produce high quality data.

Overall, it is important to know that not all HTP systems for data collection are created equal. Knowing what HTP traits are most important to the program, frequency and scale of data collection, and resources allocation will help determine which platform will be most beneficial in HTP data collection. When implemented properly, UAS are the more promising system for SRI collection in large-scale breeding programs.

## Data availability statement

The datasets presented in this study can be found in online repositories. The names of the repository/repositories and accession number(s) can be found below: https://doi.org/10.7273/000004802.

## Ethics statement

Written informed consent was obtained from the individual(s) for the publication of any identifiable images or data included in this article.

## Author contributions

The project conceptualization and development of methodology was done by AH and AC.The formal investigation, analysis, visualization, original draft preparation, and data curation was conducted by AH. The resources, review and editing, funding acquisition, and supervision was done by AC. All authors contributed to the article and approved the submitted version.
